# Spatial meta-transcriptomics reveal associations of intratumor bacteria burden with lung cancer cells showing a distinct oncogenic signature

**DOI:** 10.1136/jitc-2022-004698

**Published:** 2022-07-06

**Authors:** Abigail Wong-Rolle, Qiang Dong, Yunhua Zhu, Prajan Divakar, Jyh Liang Hor, Noemi Kedei, Madeline Wong, Desiree Tillo, Elizabeth A Conner, Arun Rajan, David S Schrump, Chengcheng Jin, Ronald N Germain, Chen Zhao

**Affiliations:** 1 Thoracic and GI Malignancies Branch, National Cancer Institute, Bethesda, Maryland, USA; 2 Lymphocyte Biology Section, Laboratory of Immune System Biology, NIAID, Bethesda, Maryland, USA; 3 Perelman School of Medicine, University of Pennsylvania, Philadelphia, Pennsylvania, USA; 4 Bioinformatics and Computational Biosciences Branch, Office of Cyber Infrastructure and Computational Biology, NIAID, Bethesda, Maryland, USA; 5 NanoString Technologies Inc, Seattle, Washington State, USA; 6 Collaborative Protein Technology Resource, NCI, Bethesda, Maryland, USA; 7 CCR Genomics Core, National Cancer Institute, Bethesda, Maryland, USA; 8 Advanced Biomedical Computational Science, Frederick National Laboratory for Cancer Research, Frederick, Maryland, USA; 9 Thoracic Surgery Branch, National Cancer Institute, Bethesda, Maryland, USA

**Keywords:** tumor microenvironment, lung neoplasms, translational medical research

## Abstract

**Background:**

The lung intratumor microbiome influences lung cancer tumorigenesis and treatment responses, but detailed data on the extent, location, and effects of microbes within lung tumors are missing, information needed for improved prognosis and treatment.

**Methods:**

To address this gap, we developed a novel spatial meta-transcriptomic method simultaneously detecting the expression level of 1,811 host genes and 3 microbe targets (bacteria, fungi, and cytomegalovirus). After rigorous validation, we analyzed the spatial meta-transcriptomic profiles of tumor cells, T cells, macrophages, other immune cells, and stroma in surgically resected tumor samples from 12 patients with early-stage lung cancer.

**Results:**

Bacterial burden was significantly higher in tumor cells compared with T cells, macrophages, other immune cells, and stroma. This burden increased from tumor-adjacent normal lung and tertiary lymphoid structures to tumor cells to the airways, suggesting that lung intratumor bacteria derive from the latter route of entry. Expression of oncogenic β-catenin was strongly correlated with bacterial burden, as were tumor histological subtypes and environmental factors.

**Conclusions:**

Intratumor bacteria were enriched with tumor cells and associated with multiple oncogenic pathways, supporting a rationale for reducing the local intratumor microbiome in lung cancer for patient benefit.

**Trial registration number:**

NCT00242723, NCT02146170.

**WHAT IS ALREADY KNOWN ON THIS TOPIC?**

- Previous studies showed dysbiosis in lung cancer and the existence of bacteria inside of tumor cells; however, the spatial distribution of the lung intratumor microbiome and interactions of these organisms with host cells in the tumor microenvironment are unknown.

**WHAT THIS STUDY ADDS?**

- Our study showed intratumor bacteria were enriched with tumor cells compared with immune cells and associated with multiple oncogenic pathways in tumor cells.
- Bacteria burden increased from tumor-adjacent normal lung and tertiary lymphoid structures to tumor cells to the airways.

**HOW THIS STUDY MIGHT AFFECT RESEARCH, PRACTICE OR POLICY?**

- The findings of our study emphasized the importance of direct interaction between tumor cells and bacteria, supporting the therapeutic potential of reducing bacteria burden in the tumor microenvironment.

## Background

Despite recent advances in treatment, lung cancer is still the leading cause of cancer-related death,^1^ pointing to the importance of achieving a deeper understanding of the complex tumor microenvironment (TME) in this disease in seeking new therapeutic options. Previous studies have documented the mutational, transcriptional, and immunological profiles of lung cancer while other investigations have examined the gut microbiome as a potential factor contributing to disease and response to treatment.^2 3^ However, very little is known about the lung intratumor microbiome, in large part due to limited investigation in the past when the prevailing belief was that lung parenchymal tissues are sterile.^4^ However, recent progress in 16S rRNA sequencing, a culture-independent bacterial analysis method, shed light on the local intratumor microbiome in lung cancer and showed that multiple bacteria species are associated with these malignancies.^5 6^

Our previous work using a genetically engineered mouse model involving transformed cells with *Kras* activation and a *Tp53* loss-of-function mutation (KP mice) found an increased lung bacterial burden in tumor-bearing mice and a reduced tumor burden when the lung bacterial microbiome was decreased by local antibiotic delivery.^7^ Other studies have highlighted the existence of bacteria within tumor cells^8^ and tumor cells have been shown to present bacterial antigens to T cells,^9^ together suggesting an important role of lung intratumor microbes in the state of the tumor cells themselves and the nature of the local immune TME. However, the spatial distribution of the lung intratumor microbiome, the extent and nature of interactions of these organisms with tumor cells, immune cells, and stromal cells, and the specific manner in which these organisms influence the TME are still uncharacterized. To address these unanswered questions, spatially resolved co-detection of bacteria and host molecular markers and transcripts is required. Traditional imaging methods, including multiplex immunohistochemistry and fluorescent *in situ* hybridization(FISH), are still limited in the extent to which they can analyze the tumor in a comprehensive and unbiased manner. Furthermore, current sequencing technologies are unable to obtain bacterial and host transcriptional information simultaneously. Here, we describe a new approach to obtaining this needed information and provide evidence that local bacterial association with tumor cells is pro-oncogenic in lung cancer.

## Methods

### Patient samples

Samples from 12 patients with early stage lung cancer who were enrolled in clinical trials NCT00242723 and NCT02146170 were used for this study. Lung samples, including adjacent normal regions, were surgically resected and formalin-fixed and paraffin embedded (FFPE).

### Mouse samples

Germ-free *Kras*^LSL-G12D/+^; *p53*^flox/flox^ (KP) mice have been described previously.^1^ Briefly, KP mice used in this study were derived by embryo transfer using sterile techniques and maintained in germ-free conditions by following stringent standard operating procedures to prevent contamination. The germ-free status was confirmed by microbiological monitoring by culture and fecal PCR, alternatively, every 2 weeks. Tumor-bearing lung lobes were fixed in 4% paraformaldehyde overnight and embedded in paraffin.

### Sample processing for NanoString Digital Spatial Profiler (DSP) analysis

FFPE blocks were sectioned onto Superfrost Plus microscope slides at 5 μm thickness. One section from an FFPE block from a tumor-bearing germ-free KP mouse was placed on each slide as an inter-slide control for the baseline of the *16S rRNA* signal. Sections from one or two patient samples were placed on the same slides, according to their size. Slides were processed for DSP tissue treatment within 1 hour of sectioning to ensure RNA quality.

For the NanoString GeoMx DSP RNA assays, slides were prepared following the Leica Biosystems BOND RX FFPE RNA Slide Preparation Protocol in the GeoMx NGS Slide Preparation User Manual (NanoString, MAN-10 115-04). Briefly, slides were baked at 60°C for 30 min and then loaded into the Leica BOND RX device. Slides were treated sequentially following Leica BOND RX default Bake & Dewax Protocol, HIER (ER2, 20 min, 95°C) Protocol and GeoMx DSP RNA Slide Prep Protocol (1 mg/mL proteinase K (Ambion, cat. 2546) in 1X phosphate-buffered saline (PBS) at 37°C for 15 min). After pretreatment, slides were hybridized with the GeoMx Cancer Transcriptome Atlas Panel (1811 targets, cat. 121400101) supplemented with customized bacterial 16srRNA-cytomegalovirus (CMV)-fungal spike-in probes provided by NanoString (detailed probe information in online supplemental table 1). One at a time, slides were dried of excess 1X PBS, set in a hybridization chamber lined with Kimwipes wetted with Diethyl pyrocarbonate(DEPC)-treated water, and covered with 200 μL prepared Probe Hybridization solution. HybriSlips (Grace Biolabs, cat. 714022) were gently applied to each slide and they were left to incubate at 37°C overnight.

10.1136/jitc-2022-004698.supp1 Supplementary data

10.1136/jitc-2022-004698.supp2 Supplementary data

After hybridization, the HybriSlips were removed by dipping the slides in 2X saline-sodium citrate (SSC) (Sigma-Aldrich, cat. S6639)/0.1% Tween-20. To remove unbound probes, the slides were washed twice in Stringent Wash (50% formamide (ThermoFisher, cat. AM9342)/2X SSC) at 37°C for 25 min, followed by two washes in 2X SSC for 2 min. Slides were blocked in 200 μL Buffer W (NanoString), placed in a humidity chamber and incubated at room temperature for 30 min. Morphology marker solution was prepared for four slides at a time: primary antibody: 20 μL CD3 (Abcam, clone: F7.2.38, cat. ab17143, RRID:AB_302587) and 780 μL Buffer W; secondary antibody: 10 μL anti-mouse Ig Alexa 532 (ThermoFisher, cat. A-11002) and 790 μL Buffer W; direct antibodies: 20 µL anti-pan-cytokeratin Alexa 488 antibody (Invitrogen, clone: AE1+AE3, cat. 53-9003-82), 20 μL CD45 morphology marker (NanoString, clone: 2B11+PD7/26, cat. 121300310), 20 μL anti-CD68 Alexa 647 (Novus Bio, clone: SPM130, cat. NBP2-34736AF647) and 740 μL Buffer W for a total volume of 200 μL/slide. Slides were dried of excess Buffer W, set in a humidity chamber, covered with morphology marker solution, and left to incubate at room temperature for 1 hour during primary and secondary antibody staining. Slides were washed with 2X SSC for 5 min twice after each staining and were immediately loaded into the GeoMx instrument.

### GeoMx DSP region of interest selection and sequencing

Eight slides containing 12 patient samples and germ-free KP mouse samples were analyzed using a NanoString GeoMx instrument as described.^10^ ^11^ Briefly, RNA probe-hybridized and antibody-stained slides were scanned with a 20X objective, collecting data using FITC/525 nm (excitation 480/28 nm, emission 516/23 nm), Cy3/568 nm (excitation 538/19 nm, emission 564/15 nm), Texas Red/615 nm (excitation 588/19 nm, emission 623/30 nm), and Cy5/666 nm (excitation 645/19 nm, emission 683/30 nm) channels. Regions of interest (ROI) were selected based on morphology and cell surface marker staining. Within each large ROI, areas of interest (AOI) were identified for RNA collection based on cell-specific surface marker staining: T cells (CD3^+^), macrophages (CD68^+^CD3^−^), other immune cells (CD45^+^CD3^−^CD68^−^), tumor cells (panCK^+^), and stroma (autofluorescence^+^panCK^−^CD45^−^). ImageJ was used to generate masks for AOI collection based on these stains. The masks were imported to the GeoMx and used to guide UV light cleavage of the barcode linkers of the prebound RNA probes in each AOI and deposition of the cleaved probes in DSP collection plates.

DSP collection plates were frozen and stored at −80°C. Plates were thawed at room temperature and libraries were prepared per manufacturer’s guidelines. Collection plates were sealed with a semi-permeable membrane and dried down at 65°C for 1 hour. Each well was resuspended in 10 µL DEPC-treated water, incubated at room temperature for 10 min, and then spun down. The library preparation was carried out in a 96-well PCR plates by mixing 2 μL PCR mix (NanoString), 4 μL of index primer mix (NanoString), and 4 μL of DSP sample. The following PCR program was used to amplify the Illumina sequencing compatible libraries; 37°C for 30 min, 50°C for 10 min, 95°C for 3 min, followed by 18 cycles of (95°C for 15 s, 65°C for 1 min, 68°C for 30 s), 68°C for 5 min and a final hold at 4°C. A total of 6 plates of 96 wells each were used.

The indexed libraries were pooled with an 8-channel pipette by combining 2 μL per well from the 12 columns (one 96-well plate) into 8-well strip tubes and then pooled into a 1.5 mL tube. The combined 50 µL pools were incubated with 60 μL SPRIselect beads (Beckman Coulter, cat. B23318) (1.2X bead to sample ratio) for 5 min in a 1.5 mL tube followed by standing on a magnetic stand for 5 min before removal of the supernatant. The beads were then washed twice with 200 µL of 80% ethanol and air dried for 3 min before being eluted with 50 µL elution buffer (10 mM Tris-HCl pH 8, 0.05% Tween-20, Teknova cat. T1485). Then a second round of SPRIselect beads (Beckman Coulter, cat. B2331860 ul; 1.2X bead to sample ratio) selection was carried out directly on the bead suspension as above. The washed beads were eluted in 20 µL elution buffer (10 mM Tris-HCl pH 8, 0.05% Tween-20, Teknova cat. T1485) and 18 µL of the supernatant was extracted to a new tube. The 18 µL of clean-up supernatant from each 96-well plate was pooled, and the library fragment size was assessed with the D1000 Tape Station assay (Agilent Technologies) and the expected size of ~162 bp was observed.

Total target counts per DSP collection plate for sequencing were calculated based on the NanoString DSP Worksheet. The target sequencing depth was 30 counts/μm^2^ for a total of 1.5B reads. Libraries were sequenced with a 100-cycle S1 kit on the NovaSeq 6000 at the CCR Sequencing Facility (Frederick, Maryland, USA).

### Expression analysis of host transcriptome, microbial *16S rRNA*, CMV *UL83*, and fungal *28S rRNA* expression calculation

Sequence reads were demultiplexed and converted to digital count conversion (DCC) files using the NanoString DnD pipeline (Supplementary Data1). DCC files were further converted to an expression count matrix. For analysis of the host transcriptome, the 75th percentile (Q3) of the target counts of each probe pool in each AOI type was calculated and normalized to the geometric mean of the 75th percentiles across all AOIs of each type to give normalization factors (Supplementary Data2). Target counts were divided by these normalization factors to give normalized expression counts. Regarding spike-in targets, all genes were normalized to negative probes in the customized probe set. To further control interslide variation, counts were further normalized by the consecutively cut germ-free KP samples. The comparison of bacterial *16S rRNA*, CMV *UL83*, and fungal *28S rRNA* expression was tested using the two-sided Wilcoxon test. Correlation analysis of *16S rRNA* signal and host transcripts was done by calculating the Spearman’s rank correlation coefficient for each host gene against the *16S rRNA* count in that sample. All normalization analyses and spatial-deconvolution were done using the NanoString DSP analysis platform. Immune cell abundance was calculated using a previously described spatial-deconvolution algorithm^12^ and reference libraries were generated based on prior studies.^13–15^ Statistical analysis was done in R Studio, and pathway analysis was done with QIAGEN Ingenuity Pathway Analysis.^16^

### RNAscope

For the RNAscope co-detection assay, staining was performed on FFPE slides according to the manufacturer’s protocol (RNAscope LS Multiplex Fluorescent Assay combined with Immunofluorescence—Integrated Co-Detection Workflow on Leica Bond RX, MK 51-152) using the RNAscope LS Multiplex Fluorescent Reagent Kit (ACD Bio, 322800) with the RNAscope 2.5 LS Probe-16S-C1 probe (ACD Bio, 1056988-C1 and the DAPI, Opal 570, Opal 650 (Akoya Biosciences, FP1488001KT, dilution: 1:1500). Freshly cut FFPE sections on slides were loaded into the Leica BOND RX. Slides were treated sequentially following the Leica BOND RX default Bake & Dewax Protocol and ACD HIER (ER2, 30 min, 95°C) protocol. Slides were stained with anti-pan-cytokeratin antibody (Leica Biosystems, clone: AE1/AE3, cat. 1 mL NCL-L-AE1/AE3-601, dilution: 1:100) for 15 min, followed by postfixation with 10% neutral buffered formalin for 30 min. Fixed slides were treated with RNAscope 2.5 LS Protease III for 30 min, and then ACD 16s rRNA pre-treatment reagent (ADC Bio, cat. 300040) for 30 min. Treated slides were hybridized with RNAscope 2.5 LS Probe-16S-C1 probe (ACD Bio, cat. 1056988-C1) for 2 hours. *16S rRNA* and pan-cytokeratin signals were amplified according to the manufacturer’s guidance. Slides were mounted with Fluoromount-G (Southern Biotech, cat. 0100-01) and examined on a Leica THUNDER Imager. Images were analyzed with Imaris software (Bitplane, RRID:SCR_007370), ImageJ (RRID:SCR_003070), and Adobe Illustrator (RRID:SCR_010279).

### β-Catenin immunohistochemistry (IHC)

FFPE slides were treated sequentially following the Leica BOND RX default Bake & Dewax Protocol, HIER (ER2, 20 min, 95°C) protocol and the immunohistochemistry (IHC) Leica BOND protocol (anti-β-catenin antibody, Cell Signaling Technology, clone: 15B8, cat. 37 447S; SignalStain Antibody Diluent, Cell Signaling Technology, cat. 8112; BOND Polymer Refine Detection, Leica Biosystems, cat. DS9800). Stained slides were treated with 95% ethanol for 10 s twice and 100% ethanol for 10 s twice, followed by xylene for 10 s twice. Slides were mounted with Fisher Chemical Permount Mounting Medium (Fisher Scientific, cat. SP15-100) and scanned on HAMAMATSU NanoZoomer 2.0RS. Images were analyzed using the HALO platform (Indica Labs).

## Results

### Spatial meta-transcriptomic analysis method

**Figure 1:**
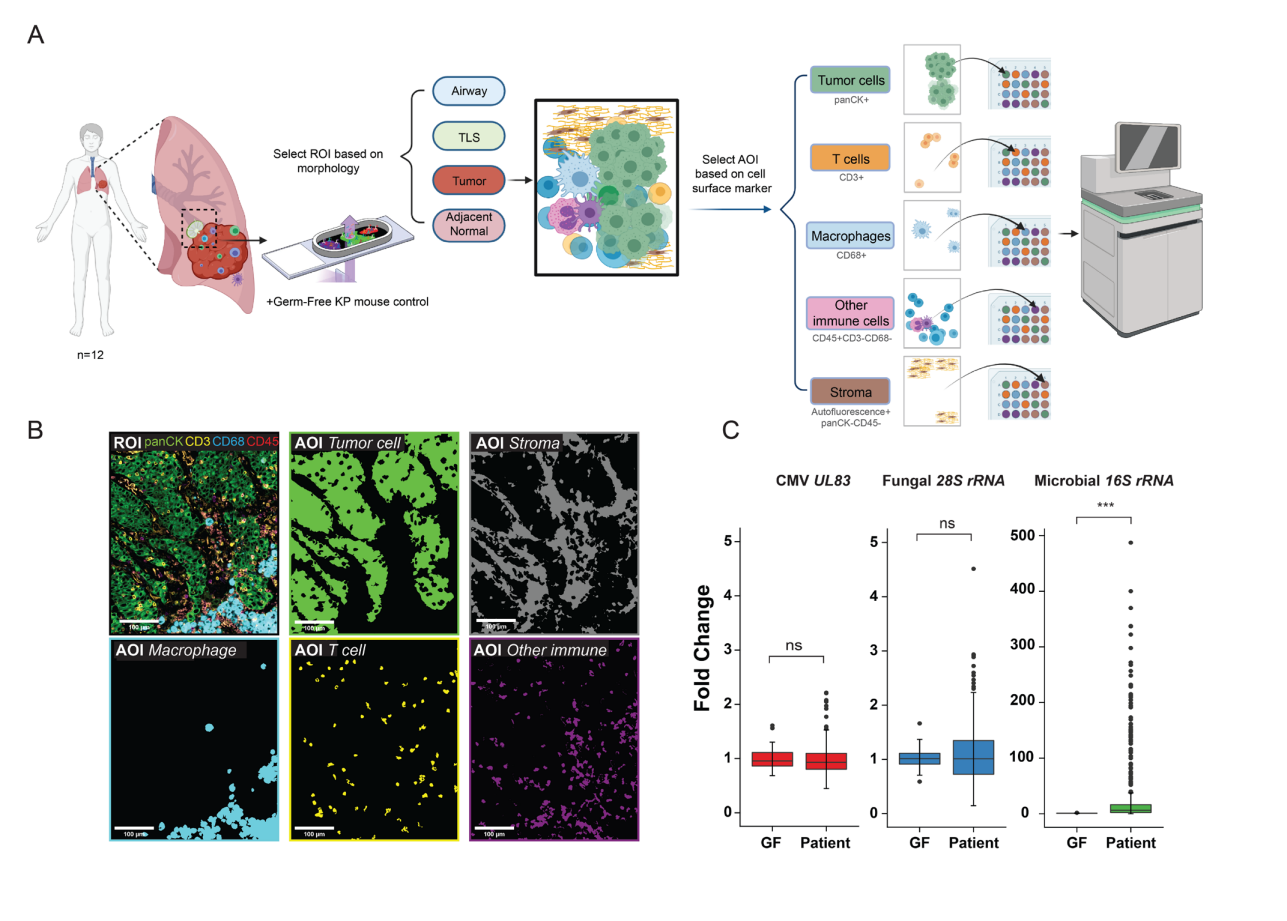
Spatial meta-transcriptomic analysis of lung cancers. (A) Study scheme: formalin-fixed, paraffin-embedded (FFPE) samples from 12 patients with lung cancers were sectioned onto 8 slides along with a consecutively sectioned germ-free mouse lung tumor sample on each slide as interslide negative control. Regions of interest (ROIs) (adjacent normal tissue n=56, tumor regions n=384, tertiary lymphoid structures (TLS) n=20, airway n=4) were selected based on morphology. Further area of interest (AOI) selection was done within these defined ROIs to investigate different cell types in the tumor microenvironment (TME), based on cell surface markers. RNAs from AOIs within each ROI were collected, separated, and sequenced as previously described. (B) Representative ROI and AOIs (tumor cells, macrophages, T cells, other immune cells, and stroma) collected from the ROI. Colored segments (AOIs) show areas collected for sequencing. (C) Cytomegalovirus (CMV), fungal, and microbial signals in germ-free KP mice tumors (GF) and patients with lung cancers (Patient) (***p<0.001, ns, not significant, t-test, error bars, SD).

In contrast to the many studies using gut (fecal) microbiome samples to examine how these intestinal bacterial species affect the growth and treatment response of various cancers, we designed our study to investigate the local microbial population within the TME itself. To simultaneously obtain local microbe and host transcriptomic data along with spatial information, we developed a novel spatial meta-transcriptomic analysis method based on the recently introduced NanoString Digital Spatial Profiler^11^ (figure 1A) that was applicable to FFPE archived tissues. As this method is a probe-based mRNA detection system, it does not require poly-A capture, allowing us to detect the abundance of bacteria (*16S rRNA*), fungi (*28S rRNA*), and CMV (*UL83*) transcripts in addition to RNA products from 1,800 human genes involved in immune and cancer pathways (online supplemental figure S1). Both host and microbe transcriptomic information was collected within microscopically defined regions of interest based on immunofluorescent imaging of specific cell types stained for cell surface markers: tumor cells (panCK^+^), T cells (CD3^+^CD45^+^), macrophages (CD68^+^CD45^+^), other immune cells (CD3^−^CD68^−^CD45^+^), and tumor stroma (autofluorescence^+^, panCK^−^CD45^−^) (figure 1B). Information from a minimum of 30 cells per cell type was collected within each ROI from each patient tumor or control tissue sample. The purity of transcriptomic information from each cell type was further confirmed by the gene expression profile (online supplemental figure S2).

10.1136/jitc-2022-004698.supp3 Supplementary data

10.1136/jitc-2022-004698.supp4 Supplementary data

Contamination from environmental bacteria is a major challenge in microbiome research, especially in studying the human microbiome in tissue samples. In addition to negative spike-in probes, we used germ-free tissue from KP mice, an ideal negative control that lacks bacteria, fungi, and CMV but has similar tumor tissue properties as our samples. Furthermore, to control for inter-measurement variation, serial sections of the same germ-free sample were used for all measurements.

### Spatial distribution of lung intratumor bacteria

**Figure 2:**
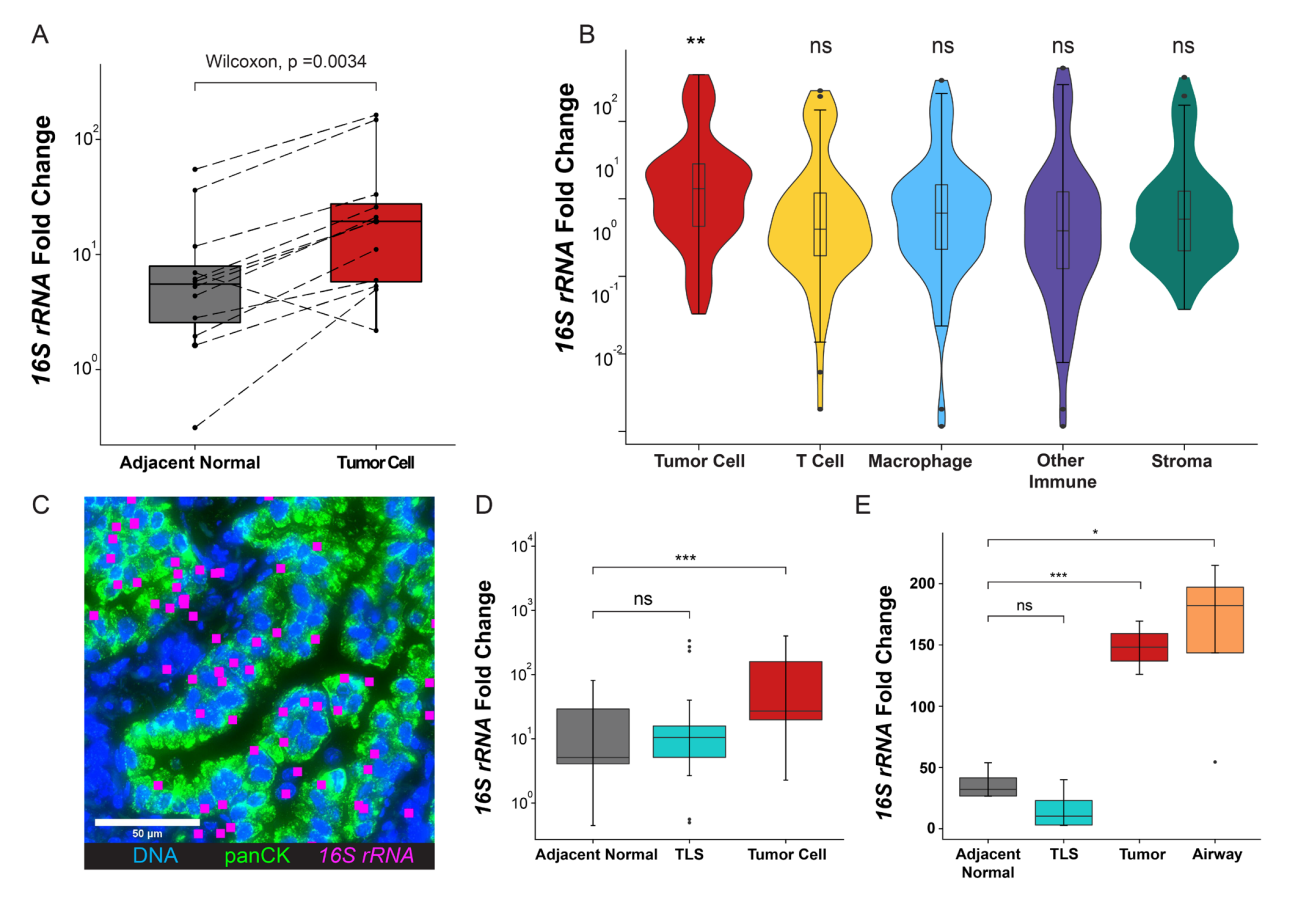
Spatial distribution of lung intratumor bacteria. (A) *16S rRNA* expression in tumor cells and adjacent normal tissue (paired two-sided Wilcoxon test). (B) *16S rRNA* expression level across different cell types in the tumor microenvironment (TME) (**p<0.01, ns, not significant, two-sided Wilcoxon test against base mean; error bars, SD). (C) Representative RNAscope image showing the spatial distribution of bacterial *16S rRNA* signal (rendered magenta squares) within tumor cells and in TME. (D) *16S rRNA* expression level in adjacent normal tissues, tertiary lymphoid structures (TLS), and tumor cells (***p<0.001, ns, not significant, one-way analysis of variance (ANOVA) test; error bars, SD). (E) *16S rRNA* expression level in adjacent normal tissues, TLS, tumor cells, and airway from one patient (*p<0.05, ***p<0.001, ns, not significant, one-way ANOVA test; error bars, SD).

Surgically resected samples from 12 patients with early stage lung cancer who were naïve to systemic therapy were analyzed (table 1). These patients did not have clinically detectable pulmonary infection and did not receive antibiotic treatment prior to surgery. Lung cancer samples showed significantly higher bacterial burden compared with control mouse germ-free samples (figure 1C); no fungal or CMV signals over background were detected in the patient samples. Therefore, we focused on intratumor bacteria in our study. Next, we studied the spatial distribution of the lung intratumor bacteria. Since post-obstructive pneumonia, an infection of the lung parenchyma secondary to obstruction caused by tumors, is a common clinical complication in patients with lung cancer,^17^ we wondered if lung intratumor bacteria were highly enriched in tumor-adjacent normal lung parenchyma. Our measurements instead revealed that the bacterial burden associated with tumor cells was much higher than present in adjacent normal lung tissue (figure 2A, online supplemental figure S3). We next compared bacterial burden among cells within the TME, specifically T cells, macrophages, other immune cells, and stroma. We examined each cell type within every individual ROI and found that although immune cells, including macrophages, neutrophils, T cells, and B cells are well known to interact with various bacteria, tumor cells harbored the highest bacterial burden in the TME (figure 2B, C). To our knowledge, this is the first quantitative data revealing that tumor cells harbor more bacteria than other cell types in the nearby TME.

10.1136/jitc-2022-004698.supp5 Supplementary data

**Table 1:** Patient characteristics

Patient	Gender	Age (years)	Staging	Histology	Smoking history	Driver mutation	Tumor size (cm)	Tumor location	Underlying pulmonary diseases
1	Male	74	IIA	Lung adenocarcinoma	Non-smoker		4.5	Peripheral	
2	Male	72	IA	Lung adenocarcinoma	Non-smoker	*EGFR (p.Glu746_Ala750del)*	1.6	Peripheral	History of community-acquired pneumonia 18 months prior to NSCLC diagnosis
3	Female	71	IA	Lung adenocarcinoma	Non-smoker	*EGFR (p.Glu746_Ala750del)*	2	Peripheral	
4	Female	60	IA	Lung adenocarcinoma	Smoker	*BRAF (p.Val600Glu)*	2	Peripheral	
5	Male	69	IA	Lung adenocarcinoma	Non-smoker	*EGFR (p.Glu746_Ala750del)*	3	Peripheral	
6	Male	62	IA	Lung squamous cell carcinoma	Smoker		3	Peripheral	
7	Male	50	IA	Lung squamous cell carcinoma	Smoker		1.2	Peripheral	
8	Female	66	Limited stage	Small cell lung cancer	Non-smoker		1.5	Peripheral	
9	Male	56	IA	Lung adenocarcinoma	Non-smoker	*EGFR (p.Glu746_Ala750del)*	1.8	Peripheral	Pulmonary embolism
10	Female	68	IA	Lung adenocarcinoma	Smoker	*KRAS (p.Gly12Cys*)	2.8	Peripheral	COPD
11	Female	78	IA	Lung adenocarcinoma	Non-smoker	*EGFR (p.Leu858Arg*)	1.6	Peripheral	
12	Male	58	IIB	Lung squamous cell carcinoma	Smoker		3.5	Central	COPD

COPD, chronic obstructive pulmonary disease; NSCLC, non-small cell lung cancer.

Tertiary lymphoid structures (TLS) play an important role in regulating local immune responses in cancer, infection, and autoimmune disease, and their presence correlates with prognosis and responses to immunotherapy in lung cancer.^18–20^ The bacterial burden in TLS and the relationship of these structures to lung intratumor bacteria were previously uncharted in lung cancer. Our data showed that tumor cells have a much higher bacterial burden compared with TLS and adjacent normal tissue (figure 2D). In one patient sample, small airway tissue was included, and we further investigated the bacterial burden among small airways, tumor cells, TLS, and adjacent normal lung tissue. Bacterial burden peaked in the airways (figure 2E), was lower in tumor cells, and further decreased in adjacent normal lung tissue and TLS. This gradient suggests that airways, rather than blood-borne bacteria that might originate in the gut, may be a source of intratumor bacteria in lung cancer.

### Effects of the lung intratumor microbiome

**Figure 3:**
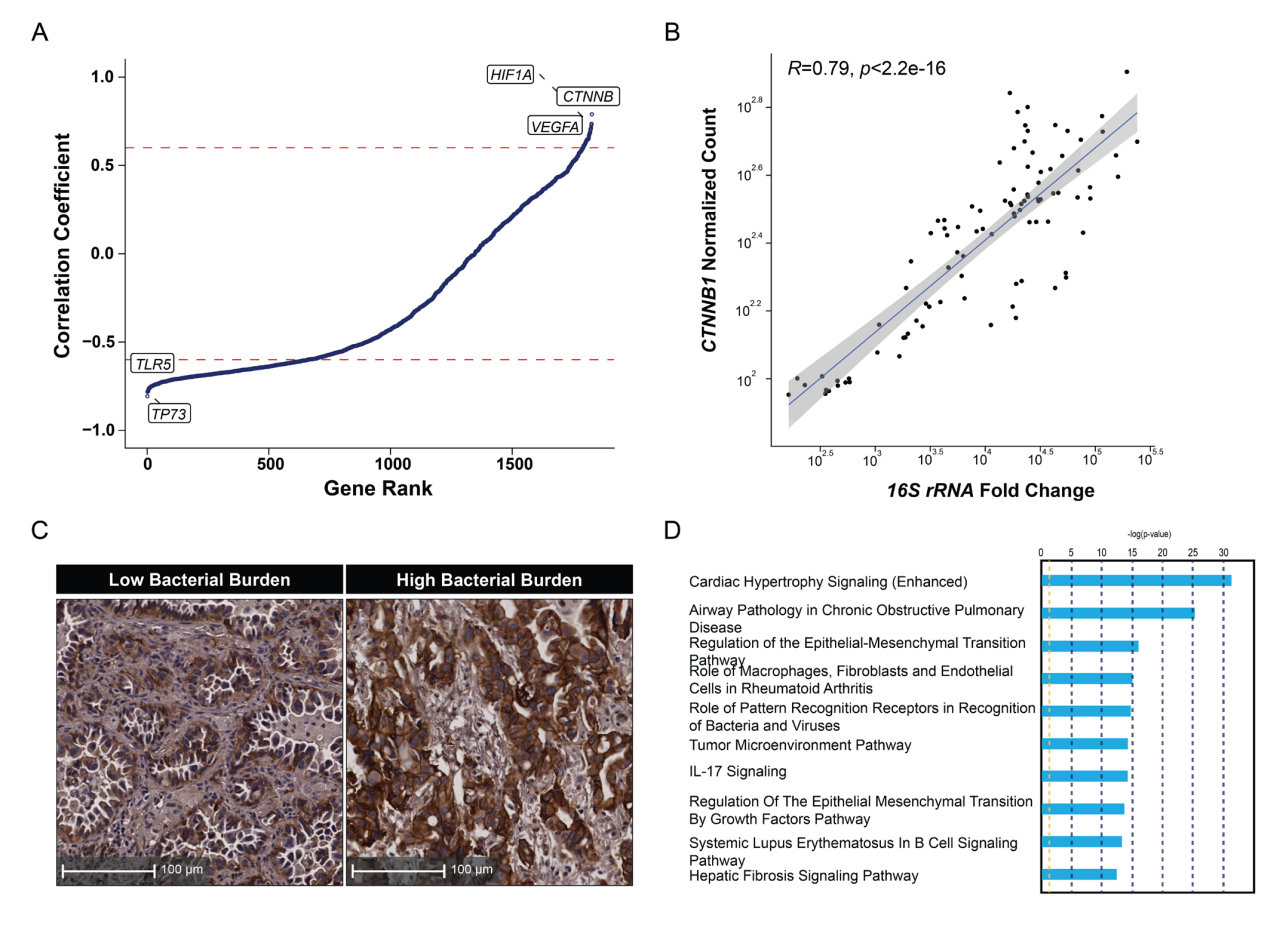
Correlations of lung intratumor bacterial burden with tumor cells’ function. (A) Correlation of host genes with bacterial burden in tumor cells (Spearman’s rank correlation, R=0.7 cut-off shown in dotted red line). (B) Correlation between host *CTNNB1* expression and bacterial burden in tumor cells (Spearman’s rank correlation, 95% CI shown in gray). (C) Representative images of immunohistochemistry staining of β-catenin in tumor regions with known low or high bacterial burden. (D) Ingenuity pathway analysis of genes correlated with bacterial burden in tumor cells.

After identifying the spatial distribution of the lung intratumor microbiome, we turned to an analysis of the effects of tumor cell-associated bacteria on the state of the malignant cells, analyzing the correlation between gene expression and bacterial burden in tumor cells (figure 3A). Genes involved in the Wnt/β-catenin (*CTNNB1),* hypoxia (*HIF1A*),^21^ and angiogenesis (*VEGFA*) pathways showed a strong positive correlation with bacterial burden, while genes affecting cell cycle (*TP73*) and pattern recognition (*TLR5*)^22^ had negative correlations. β-Catenin had the strongest correlation with bacterial burden (figure 3B), and IHC confirmed this quantitative association (figure 3C). These findings suggest that intratumor cell bacteria in lung cancer is correlated with tumor growth^23^ and an immune-resistant TME^24^ via the β-catenin pathway. Pathway analysis of tumor genes whose expression correlated with bacterial burden also identified pathways involving cell growth and epithelial-mesenchymal transitions (figure 3D), again pointing to a protumor effect of these bacteria. However, genes encoding components of the antigen presentation pathway including HLA molecules, key components typically downregulated during tumor immuno-evasion, did not show a strong correlation with bacterial burden. These data suggest that intratumor cell bacteria may not generally increase the immunogenicity of tumor cells, in contrast to a recent report^9^ (online supplemental figure S4); if this were the dominant effect in the TME, an increase in T cell recognition due to presentation of foreign microbial antigens might be expected to produce an immuno-selection signature in growing tumors, but this was not observed. Within the TME, bacterial burden showed weak correlations with the phosphoinositide 3-kinase (PI3K)/AKT and mammalian target of rapamycin pathways in T cells, the hypoxia-inducible factor 1-alpha pathway in macrophages, the complement system in other immune cells, and the PI3K/AKT pathway in stromal cells (online supplemental figure S5).

10.1136/jitc-2022-004698.supp6 Supplementary data

10.1136/jitc-2022-004698.supp7 Supplementary data

**Figure 4:**
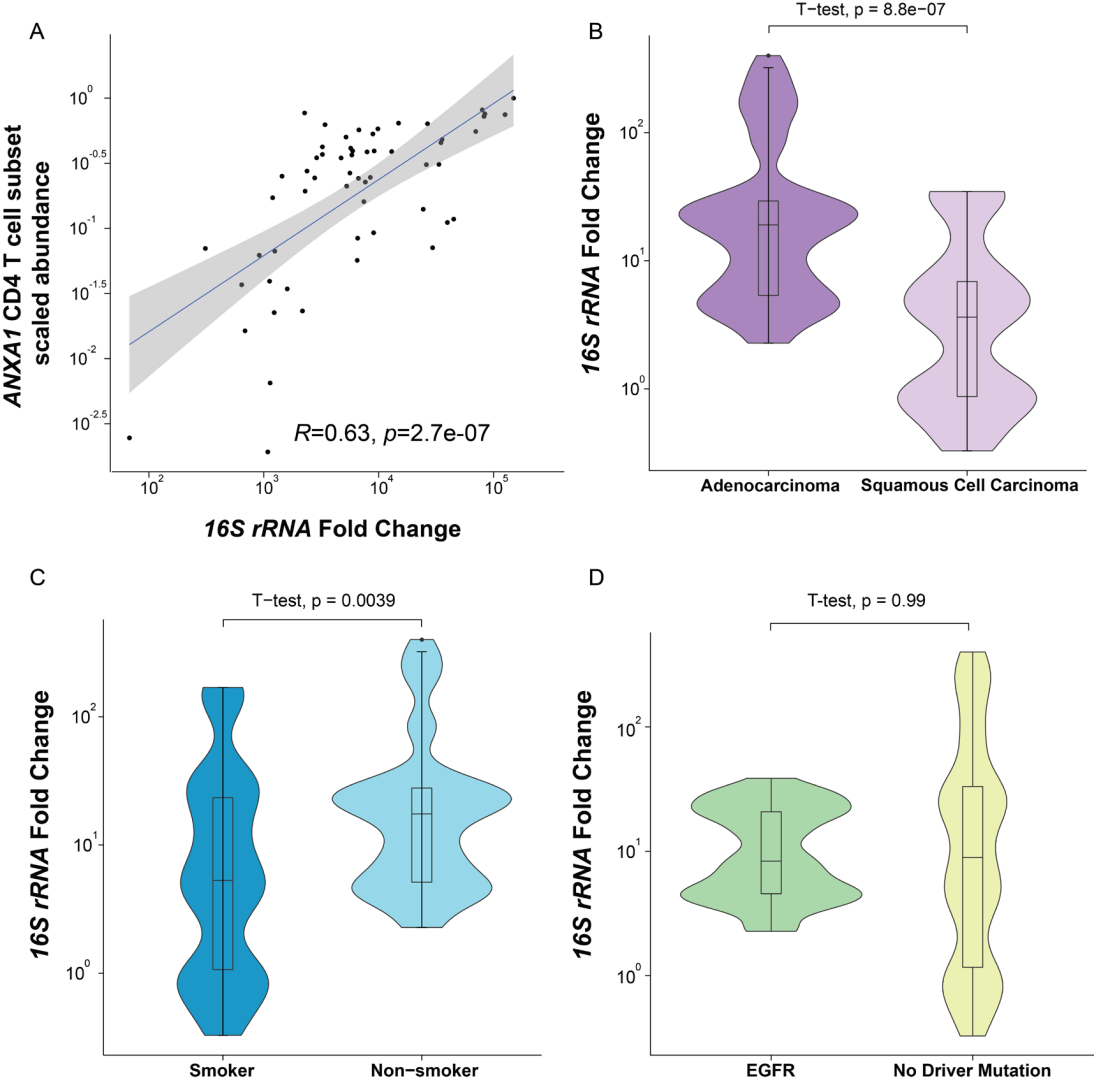
Association of bacterial burden with immune cell abundance, histology type, patient history, and mutation profile. (A) Correlation between bacterial burden and the scaled abundance of *ANXA1* CD4 T cell subset (Spearman’s rank correlation, 95% CI shown in gray). (B) Bacterial burden in tumor cells with different lung cancer histology subtypes (two-sided t-test). (C) Bacterial burden in tumor cells from patients with different smoking histories (two-sided t-test). (D) Bacterial burden in tumor cells with different driver mutation status (Spearman’s rank correlation, 95% CI shown in gray).

Despite the lower bacteria burden in non-tumor cells in the TME, we also conducted an analysis of the correlation of immune or stromal subgroups with bacterial burden via deconvolution of transcriptomic information from each non-tumor cell group based on previous single-cell RNA sequencing results.^12–15^ We discovered a correlation between the abundance of CD4 T cells with the bacterial burden, more specifically a subgroup of conventional (non-regulatory αβ) CD4 T cells with *ANXA1* gene expression^14^ (figure 4A, online supplemental figure S6).

10.1136/jitc-2022-004698.supp8 Supplementary data

To explore other factors that may affect lung intratumor bacteria, we studied the relationship between bacterial burden and tumor intrinsic factors, such as histology subtypes and mutation profiles, as well as extrinsic factors such as smoking. Adenocarcinomas had a higher bacterial burden compared with squamous cell tumors (figure 4B) and the bacterial burden in tumor cells of non-smokers was higher than in smokers (figure 4C), possibly due to a hostile antimicrobial environment created by smoking. Patients with an *EGFR* mutation, a mutation observed in 12%–47% of adenocarcinomas,^25^ did not show a significant difference in bacterial burden compared with patients without a driver mutation (figure 4D).

## Discussion

Here, we present results from a new spatial meta-transcriptomic approach that provide quantitative insight into the landscape of the intratumor microbiome in lung cancer. Our analysis is distinct from prior work focused on the gut microbiome and the effects of this large microbial population on immune tone or response to therapy.^6 26^ This detailed interrogation of the local tumor environment reveals a clear enrichment of bacteria in tumor cells compared with other cell types in the TME. In addition to spatial information, we report a strong link between tumor cell-associated bacteria and positive modulation of oncogenic signaling, which traditional sequencing methods are unable to capture. Our findings highlight the potential role of lung intratumor bacteria in tumor growth. A recent study showed that epitopes from intratumor cell bacteria can be presented by tumor cells and elicit local immune responses,^9^ which can lead to immune selection of tumor cells.^27^ However, we did not observe a robust correlation between high bacterial burden and low major histocompatibility complex class I expression, commonly associated with immune escape in TME. The impact of intratumor cell bacteria on the antigenicity of tumor cells, however, is complex and depends on the properties of specific bacterial epitopes, which can be associated with different immune responses. Due to both the size of our study and the inability of present methods to type the bacteria in tissues at a species level, our findings provide strong correlative information but underlying causal relationships remain to be defined. Further studies are needed to discover detailed mechanisms and dissect the effects of each intratumor cell bacterial species on the function and antigenicity of tumor cells.

Peak bacterial burden was found in the airway, lower in the tumor and further reduced in adjacent normal lung. This gradient of bacterial burden suggests that intratumor bacteria in lung cancer may come from the lower airway and hence, traditional analysis of the gut microbiome, while potentially informing about overall host immune tone or competence, may miss key microbial effects occurring within lung malignancies. Additionally, the lower bacterial burden in smokers suggests that smoking may create an inhospitable environment for bacteria in general. Smoking promotes local inflammation and has highly complex effects on immune responses depending on multiple factors including smoking history, types of cells, and genetic susceptibilities. The lower bacterial burden in lung cancer patients with smoking histories could be related to increased levels of immunoglobulins in bronchoalveolar fluid.^28^

Our study has several limitations. First, it was limited to measuring universal 16S rRNA signals (total bacterial burden) and lacked bacterial species information due to the design of available probes. Prior sequencing-based studies have shown decreased bacterial α-diversity in smokers,^6^ which raises the question of whether some specific bacterial strains are enriched in smokers. Such considerations indicate a need for investigation of quantitative changes in individual bacterial species in the context of lung cancer, which will require future spatial-meta-transcriptomic technology that enables acquisition of both 16S rRNA sequence and spatial information *in situ*. Second, patients enrolled in our study were mainly with early-stage lung cancer with few underlying lung comorbidities. Further study with larger patient cohorts will be needed to investigate the correlation between intratumor bacteria and patients’ disease status, pathological features, underlying pulmonary diseases, treatment responses, and prognosis.

In conclusion, these data reveal the spatial distribution of local intratumor microbiota in lung cancer and its correlation with signals contributing to tumor growth, consistent with past implications from model systems that there may be potential therapeutic value in reducing local bacterial burden in patients with lung cancer. It also lays the groundwork for future clinical studies to investigate the effects of the intratumor microbiome in lung cancer and other cancer types by providing a novel spatial meta-transcriptomic method that captures both microbial and host transcriptome information.

10.1136/jitc-2022-004698.supp9 Supplementary data

10.1136/jitc-2022-004698.supp10 Supplementary data

## Data Availability

Data are available on reasonable request. The data that support the findings of this study are available from the corresponding author on request.
