# Supplementary material for: Spatial meta-transcriptomics reveal associations of intratumor bacteria burden with lung cancer cells showing a distinct oncogenic signature
Source: J Immunother Cancer. 2022 Jul 6;10(7):e004698. doi: 10.1136/jitc-2022-004698 (PMC9260850; doi:10.1136/jitc-2022-004698)
Supplement: Supplementary data [file jitc-2022-004698supp001.pdf]

Supplementary information

Supplementary tables:

Supplementary Table 1: Probe information

Supplementary data:

Supplementary data 1: nanoString DSP-DCC file

Supplementary data 2: nanoString DSP-Annotation file

Supplementary figure legends:

Figure S1: Validation of probes targeting CMV UL83, fungal 28S rRNA and bacterial 16S rRNA

Samples with known CMV pneumonia, skin fungal infection and lung cancer were used to validate probes targeting non-host transcripts. (*$p < 0.05$, **$p < 0.01$, ***$p < 0.001$, ns: not significant, two-sided Wilcoxon test, error bars, SD)

Figure S2: Expression level of cell type-specific genes in different AOI types

The expression of *CD3D* (T cell), *CD68* (macrophage) and *PTPRC* (all immune cells) were compared among different AOIs defined by antibody staining of cell surface markers. (*$p < 0.05$, **$p < 0.01$, ***$p < 0.001$, ns: not significant, two-sided Wilcoxon test against relevant cell type, error bars, SD)

Figure S3: Bacterial burden of tumor and adjacent normal tissue for each patient

(two-sided Student t-test, error bars, SD)

Figure S4: Correlation of HLA genes and bacterial burden in tumor cells

(Spearman's rank correlation, R=0.7 cutoff shown in dotted red line)

Figure S5: Correlation of host genes and bacterial burden in all cell types in the TME

(Spearman's rank correlation, R=0.7 cutoff shown in dotted red line)

Figure S6: Correlation between CD4 T cell scaled abundance and bacterial burden

(Spearman's rank correlation, 95% confidence interval shown in gray)
