# Supplementary figures and images for: Spatial meta-transcriptomics reveal associations of intratumor bacteria burden with lung cancer cells showing a distinct oncogenic signature
Source: J Immunother Cancer. 2022 Jul 6;10(7):e004698. doi: 10.1136/jitc-2022-004698 (PMC9260850; doi:10.1136/jitc-2022-004698)

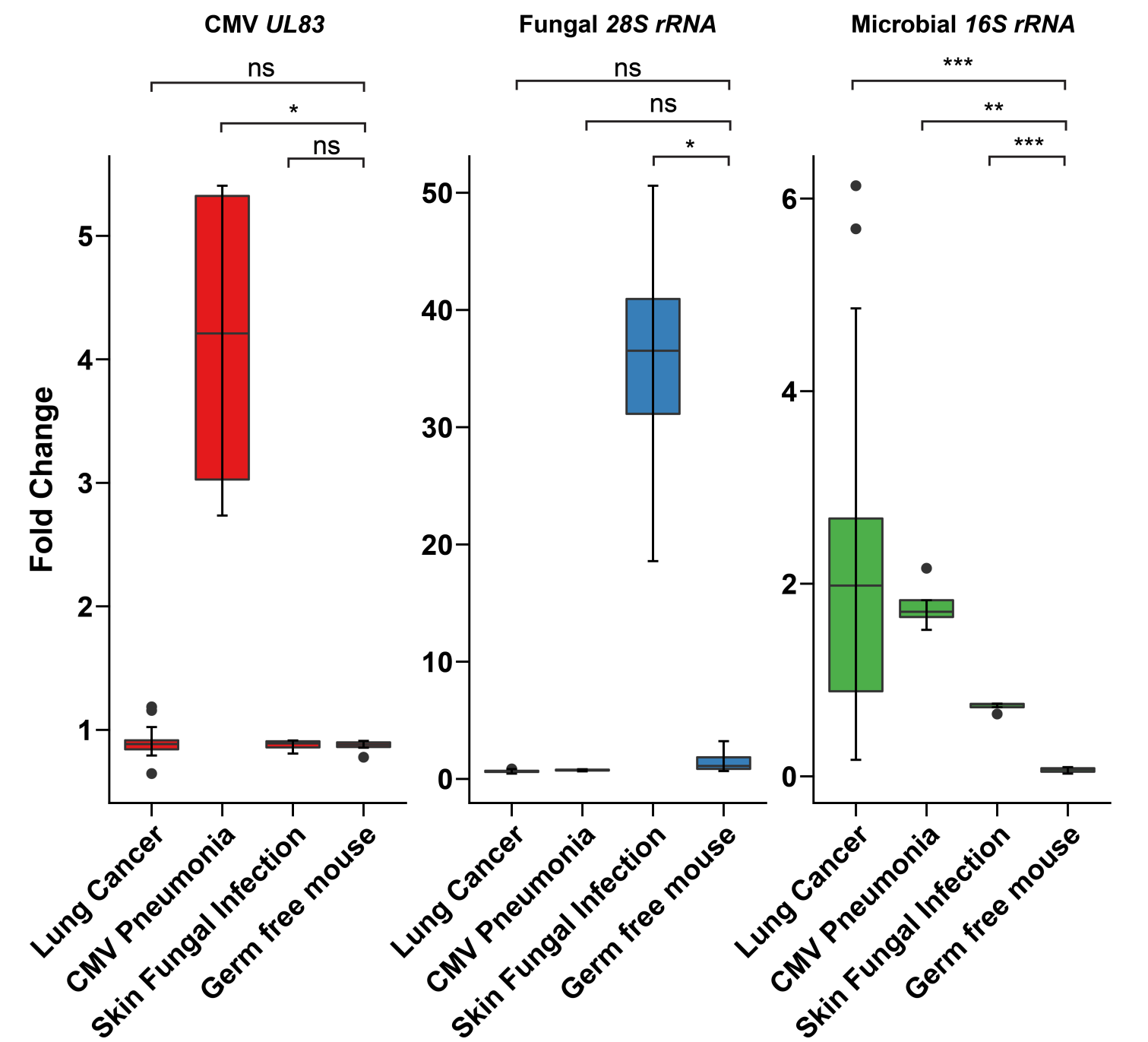

Supplement: Supplementary data [file jitc-2022-004698supp003.pdf]

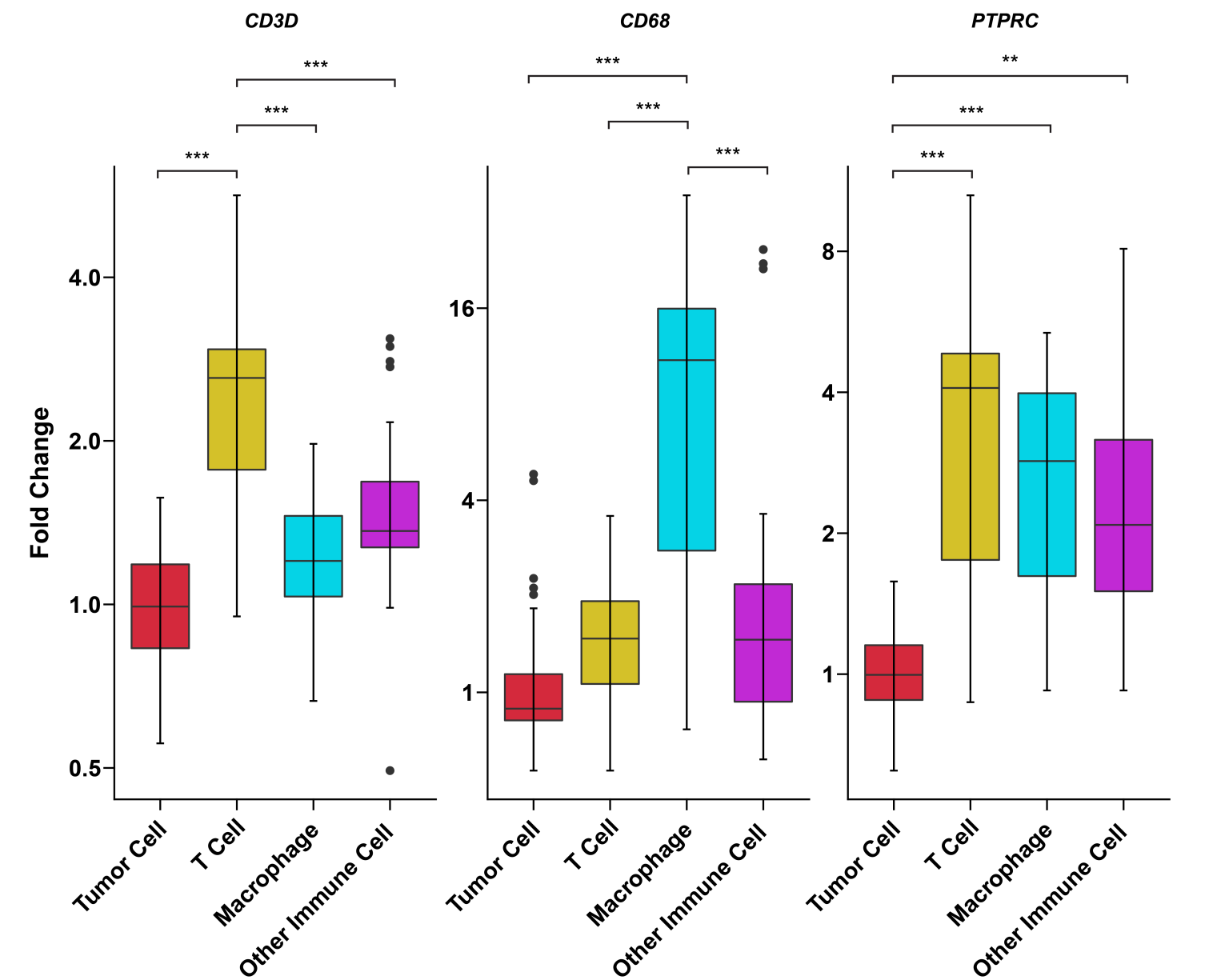

Supplement: Supplementary data [file jitc-2022-004698supp004.pdf]

Bacterial Burden of Tumor and Normal Tissue for Each Patient

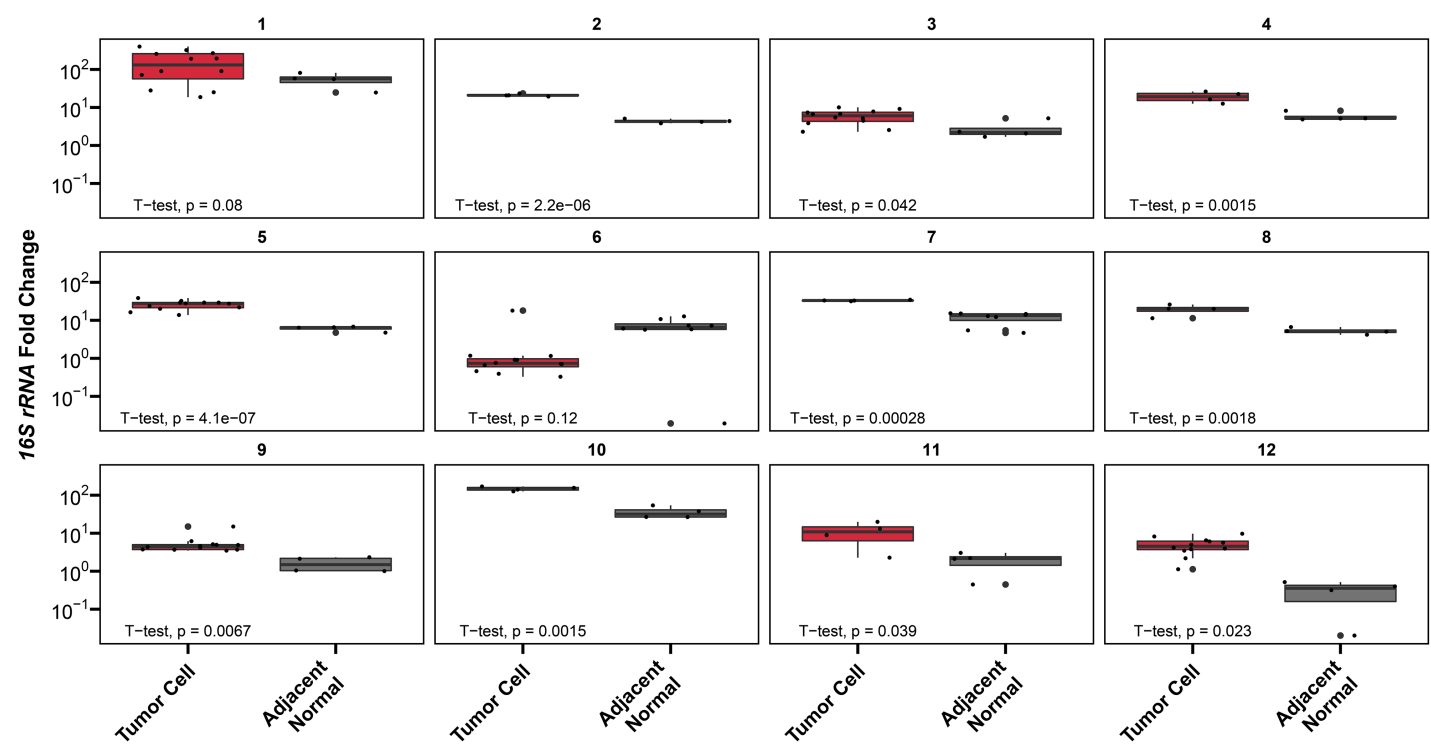

Supplement: Supplementary data [file jitc-2022-004698supp005.pdf]

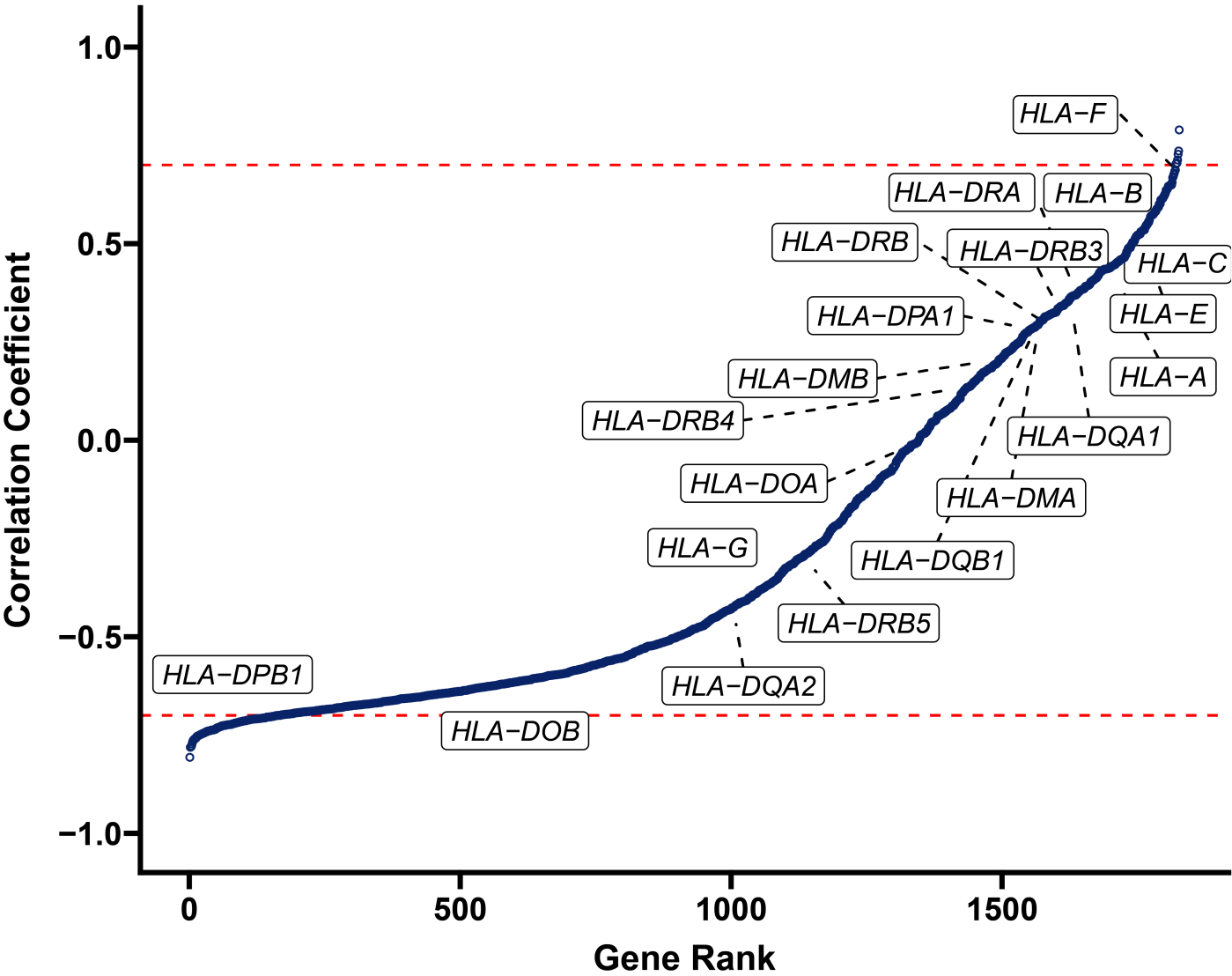

Supplement: Supplementary data [file jitc-2022-004698supp006.pdf]

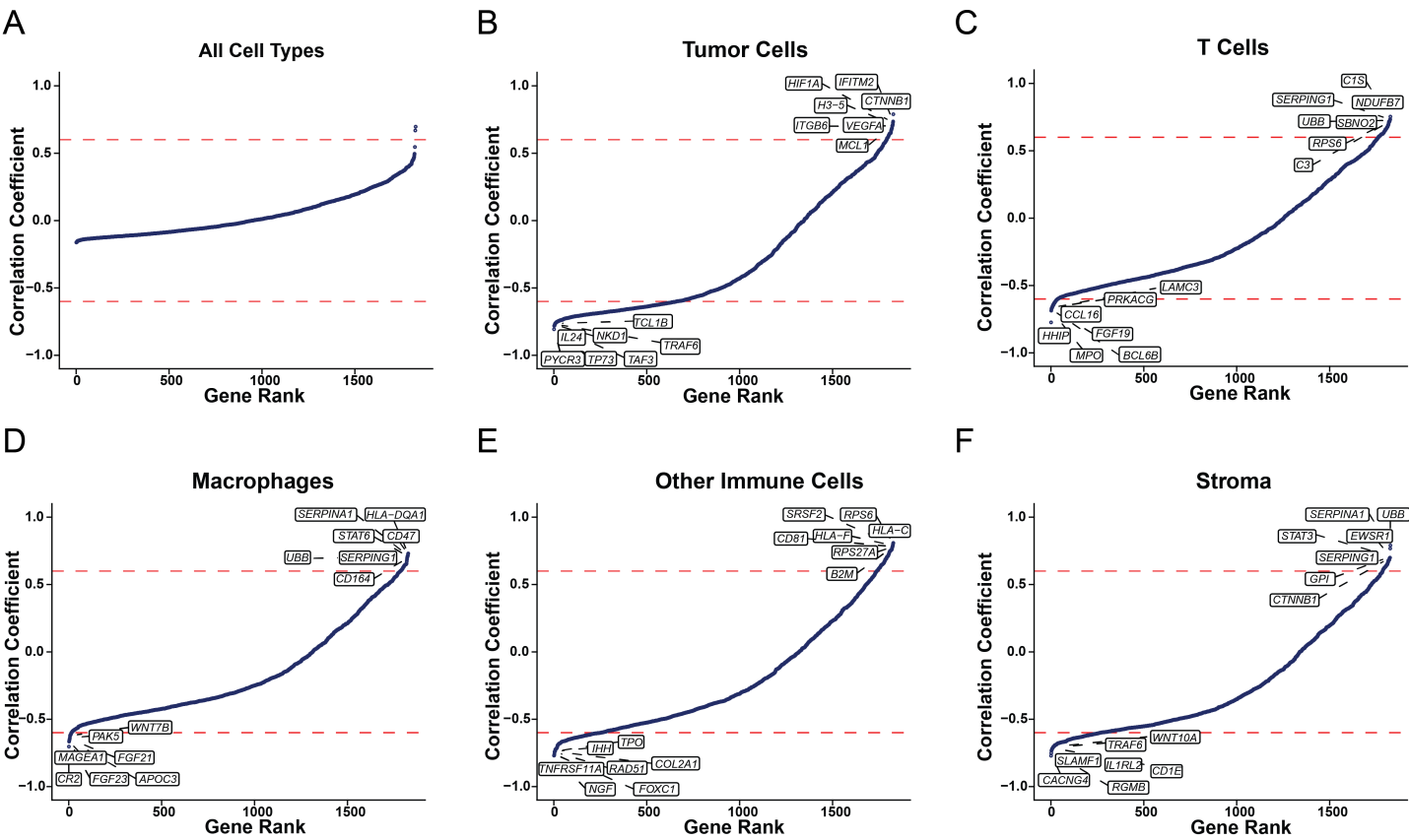

Supplement: Supplementary data [file jitc-2022-004698supp007.pdf]

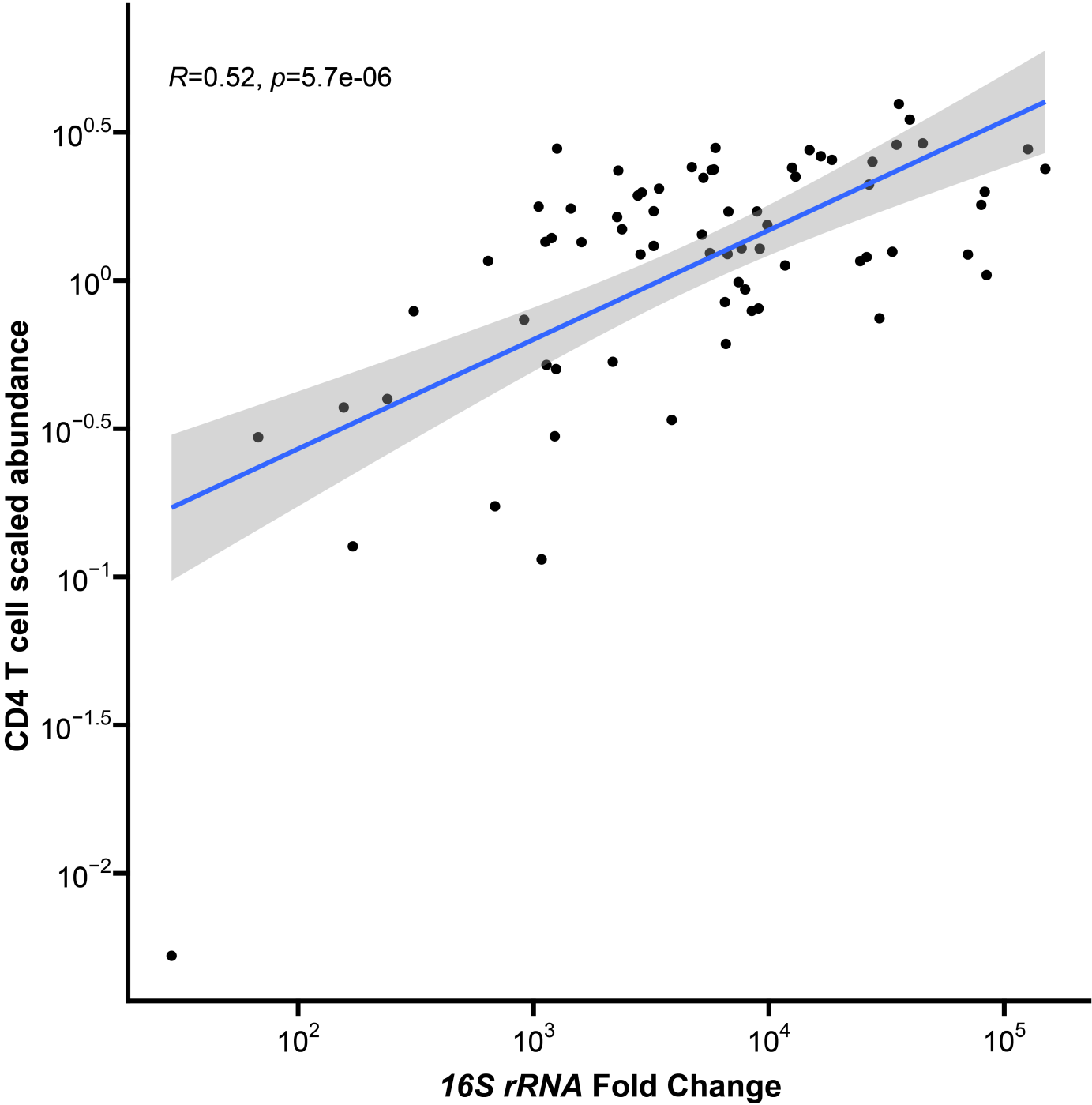

Supplement: Supplementary data [file jitc-2022-004698supp008.pdf]
